# Down-Classification of Hepatitis C Virus Diagnostics: Implications for Screening and Diagnosis

**DOI:** 10.1093/infdis/jiad299

**Published:** 2023-09-22

**Authors:** Norah Terrault

**Affiliations:** Division of Gastrointestinal and Liver Diseases, University of Southern California, Los Angeles

**Keywords:** anti-HCV, elimination, HCV RNA, point-of-care, screening

## Abstract

In November 2021, the United States Food and Drug Administration reclassified 2 types of hepatitis C virus (HCV) diagnostic tests (HCV antibody and HCV nucleic acid) from class III to class II, providing a less burdensome pathway to market for diagnostic companies. This down-classification is anticipated to facilitate innovation in HCV diagnostics, particularly for new point-of-care viral detection assays, and ultimately support HCV elimination efforts by increasing the ease of screening as well as test-and-treat models of HCV care.

An estimated 58 million people worldwide are living with hepatitis C virus (HCV) infection (as of 2019), yet only 21% (15.2 million) are aware of their diagnosis [1]. Even in the United States (US), where testing is more accessible than in many other countries, the proportion undiagnosed remains significant, estimated to be approximately 50% based on data from 2013 to 2016 [2]. This lack of awareness is one of the factors contributing to the >15 000 deaths from HCV-related complications annually in the US. In a cohort study based on analysis of electronic health records and administrative data of about 2.7 million patients visiting the same healthcare systems during 2014–2016, 21.4% of those newly diagnosed with HCV infection had late diagnosis, defined by presence of cirrhosis or cirrhosis complication within one year of diagnosis, despite many years of in-system care [3]. The coronavirus disease 2019 (COVID-19) pandemic has contributed to decreased HCV testing evident across healthcare settings [4, 5]. To get the US on track to HCV elimination will require major changes in how persons with HCV infection are identified and treated. The recently released National Strategic Plan for HCV Elimination is a call to action, with diagnostics front and center to the plan. Indeed, the pandemic has illustrated the potential to respond rapidly to an infectious crisis; the speed with which point-of-care (POC) diagnostics were developed and operationalized during the pandemic provides hope and impetus to make the same advances for HCV diagnostics.

In 2020, the US Preventive Services Taskforce recommended screening of all adults aged 18–70 years [6] and acknowledged the need to shift away from risk-based screening if unaware persons are to be successfully identified. The currently recommend diagnostic algorithm is testing for antibody to HCV (anti-HCV) followed by HCV RNA testing among those who are anti-HCV positive [7]. Societal guidelines highlight the need for repeat screening in certain populations, such as with every pregnancy, persons who use drugs, and human immunodeficiency virus (HIV)–infected men who have unprotected sex with men. Other settings that may warrant special attention for screening due to high prevalence and potential for reinfection include those in the judicial system and those unsheltered. While screening in clinics and hospitals using traditional laboratory-based screening tests will be useful for screening for most persons who are insured, alternative approaches to screening in nontraditional settings is needed to reach those at risk but who intersect less with healthcare systems. For these settings, POC testing offers significant benefits. With the view to enhancing progress in diagnosis, the first step in the HCV care cascade and entry point for persons infected with HCV to get access to curative therapy, diagnostics will need to be accessible, acceptable, and inexpensive. With increasing emphasis on “test and treat” models of care for HCV, the need for POC testing using rapid diagnostic tests has never been greater.

## REGULATORY HURDLES IN HCV DIAGNOSTICS AND THE IMPORTANCE OF FDA DOWN-CLASSIFICATION

**Figure 1.**
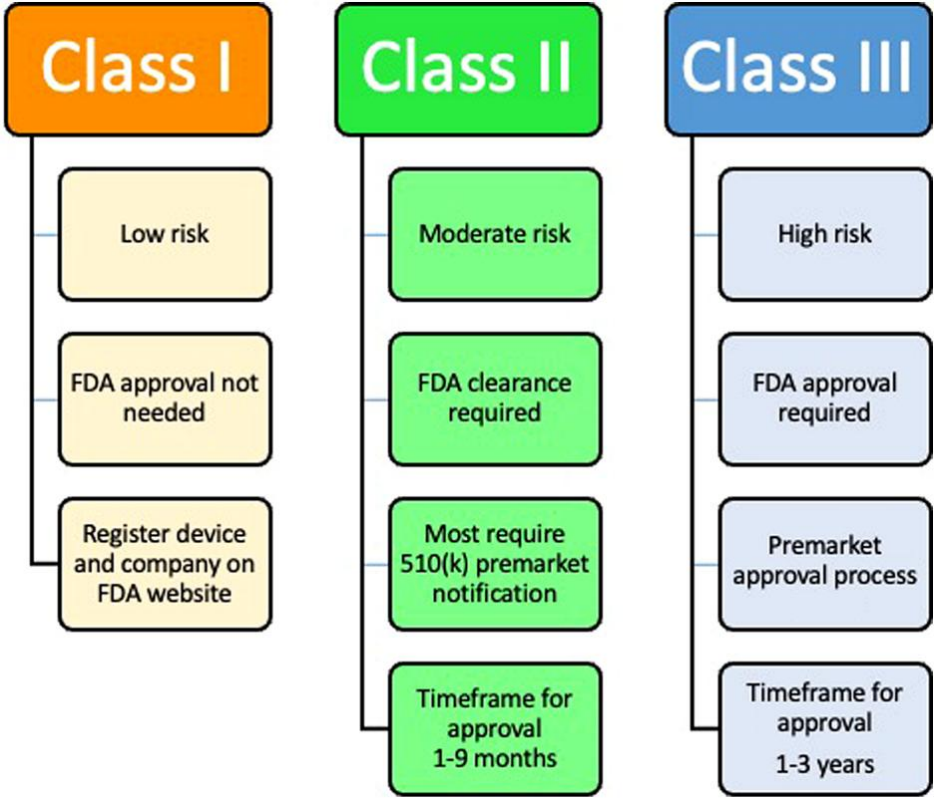
Approval process for hepatitis C virus diagnostics. Abbreviation: FDA, United States Food and Drug Administration.

The Federal Food, Drug, and Cosmetic Act has a comprehensive system for the regulation of medical devices intended for human use. There are 3 classes of devices that differ in the regulatory control needed to assure reasonable safety and effectiveness: class I (general controls), class II (general and special controls), and class III (general controls and premarket approval). Class III is the most rigorous type of US Food and Drug Administration (FDA) medical device review as it requires the submission of a premarket approval application (PMA). The currently approved HCV antibody and HCV RNA tests were required to meet this class III level of safety and effectiveness. The lengthy and arduous process for approval for class III tests has been viewed as a barrier to diagnostic development, as the typical timeline for gaining approval is measured in years rather than months (Figure 1).

Beginning in 2018, FDA, working with expert advisory panels, sought to assess the potential risks, safety, effectiveness, and benefits of reclassification of diagnostic tests from class III to class II. This work met with success, when on 19 November 2021 the FDA issued final orders reclassifying 2 types of HCV diagnostic tests from class III to class II, thereby allowing manufacturers to seek marketing clearance for diagnostic tests through a less burdensome pathway. Two types of HCV diagnostic tests were reclassified: (1) nucleic acid–based HCV RNA devices intended for the qualitative or quantitative detection of HCV RNA, and (2) certain HCV antibody devices intended for the qualitative detection of HCV antibodies [8]. The important distinction between the class II and class III pathway to approval is that class II devices can use a premarket notification 510(k) pathway, rather than submitting a PMA. The 510(k) pathway requires the demonstration of *substantial* equivalence to another legally US marketed device. Substantial equivalence means that the new device is as safe and effective as the approved device—in this case, the approved anti-HCV and HCV RNA tests. In assessing devices for the 501(k) pathway of approval, the FDA must establish that the new and predicate devices have the same intended use and that any differences between the specific technologic aspects of the tests do not raise new questions of safety and effectiveness. Then, the FDA reviews the scientific methods used to evaluate differences in technological characteristics and performance data, with the latter including clinical data, nonclinical bench data, and biocompatibility evaluation, among other data. Of note, for HCV diagnostics, the FDA does require that the test be evaluated in special controls, specifically requiring cross-reactivity studies that include samples from HCV RNA–negative subjects with other causes of liver disease, including alcohol-associated liver disease, chronic hepatitis B, and nonalcoholic fatty liver, and to provide a reasonable assurance of the safety and effectiveness of the tests.

**Figure 2.**
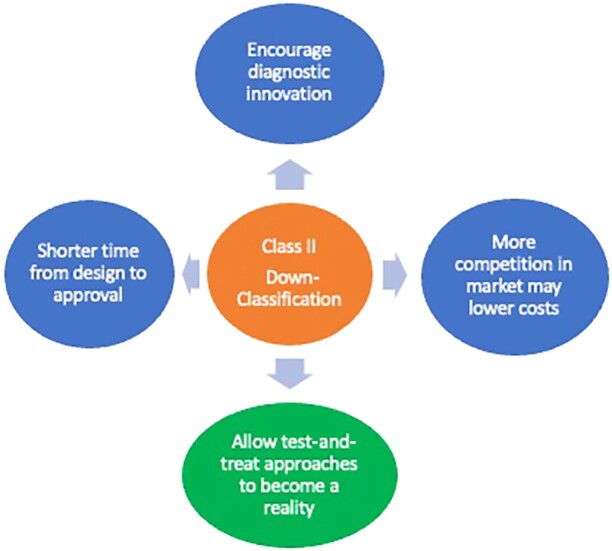
Benefits of reclassification of HCV diagnostic tests. Potential benefits of the FDA's down-classification of HCV tests from Class III to Class II may encourage more diagnostic innovation, speed up the timeline from design to approval, lower costs if more diagnostic tests come into the market and ultimately facilitate the “test-and-treat” approach to HCV care that is necessary for the achievement of HCV elimination.

While it is yet to be proven, given the “newness” of this reclassification, the anticipated benefits of this reclassification may be numerous. First, it is expected to facilitate bringing new diagnostics (anti-HCV and HCV RNA tests) to the market more quickly. Second, this may encourage innovation leading to new POC viral detection assays and simplification of diagnostic algorithms for HCV infection. Third, with more assays (and competition in this space), HCV tests of lower costs can be anticipated, which will allow their use in more resource-constrained settings (Figure 2).

The FDA emphasizes the necessity of HCV RNA test manufacturers providing a reasonable assurance of safety and effectiveness of any new device. Three health-related risks to use of nucleic acid–based tests were highlighted by the FDA: (1) inaccurate interpretation of test results, (2) failure of the device to perform as indicated, and (3) decreased test sensitivity and/or an increased rate of false-negative test reporting [8]. Mitigation strategies (labeling warnings, results interpretation information, certain design verification or validation information, analytic performance criteria, and others) can address these risks to ensure that the new diagnostic test meets the FDA's standards. For diagnostic tests for POC use, special controls that include clinical testing in the target population is important. Additionally, the FDA emphasizes that HCV RNA tests can be used for diagnosis in specified populations without prior evidence of HCV antibodies, thereby facilitating a 1-step diagnostic assay. Down-classification, per se, does not dictate the parameters of the test's performance characteristics but the diagnostic indication may.

## LIMITATIONS OF CURRENT SCREENING ALGORITHMS AND HOW DOWN-CLASSIFICATION MAY LEAD TO CHANGE

Down-classification may lead to simplified diagnostic algorithms by encouraging development of 1-step diagnostic tests. Current guidelines of HCV screening recommend testing for anti-HCV followed by confirmation of viremia using an HCV RNA test. The limitations of this 2-step approach to HCV diagnosis are well-recognized, with a significant “loss to follow-up” between antibody screening and HCV RNA confirmatory testing as high as 47% in some US studies [9]. An additional limitation of traditional laboratory testing relates to access—distance to testing locations can be barriers (including those living in remote and rural locations); in some communities, concern exists regarding stigma of being tested or found positive; and traditional laboratories can be an intimidating environment for individuals who are marginalized. The barriers to engagement in testing, such as distance, cost, and stigma, coupled with the need for 2-step testing, has spurred advocacy for a simplified testing algorithm. To reduce the risk of incomplete testing, the Centers for Disease Control and Prevention (CDC) recently recommended the adoption of a single-visit sample collection, with automatic HCV RNA testing done on anti-HCV-positive samples [10].

As previously highlighted, under the new reclassification rules, the FDA will consider approval of nucleic acid–based HCV RNA tests for use as first tests for the diagnosis of HCV infection without prior evidence of HCV antibodies. Specific populations/settings where HCV RNA as the first diagnostic test may be appropriate include populations with a high prevalence of anti-HCV, such as persons who inject drugs or persons who are justice-involved. The value of HCV RNA testing as the first diagnostic test in these settings is 2-fold. First, screening using HCV RNA tests reduces the window period for detection of new infection, as HCV RNA is detectable within 2 weeks after exposure, whereas development of anti-HCV occurs by 4–8 weeks later, on average [11, 12]. Second, it maximizes the test-and-treat opportunity and increases the likelihood of infected persons initiating HCV treatment [13].

## IMPLICATIONS OF THE DOWN-CLASSIFICATION OF HCV DIAGNOSTIC ASSAYS

Certainly, down-classification simplifies the process for diagnostic companies to get tests approved for clinical use, and it is hoped that this will encourage more innovation that ultimately will improve the ease of screening—but down-classification per se does not guarantee this.

It is well recognized that integration of HCV screening and treatment into settings where persons with HCV infection receive their primary care is the only means by which HCV elimination can be achieved. Thus, while current laboratory methods and approved HCV diagnostics will continue to play an important role in screening the population, testing in nontraditional settings is critical.

To provide HCV care in nontraditional settings, rapid diagnostic tests are needed to establish presence of infection and viremia. POC HCV RNA diagnostic tests, such as the GenXpert HCV RNA assay, are of particular interest in settings where access to traditional laboratory facilities is limited. POC diagnostics for HIV, for example, have led to increased screening rates and facilitated earlier detection of HIV. Similar outcomes with POC testing among patient populations with high HCV prevalence, such as in persons receiving opioid substitution therapy, would be highly desirable. POC HCV RNA testing is more expensive than POC HCV antibody testing and has a longer time to result—all factors that influence their use in nontraditional settings—but new technology platforms and next-generation tests that can offer equivalent accuracy with shorter turnaround time would be game-changing. Moreover, strategies to provide quality assurance to POC testing sites have been established. Identifying and validating sample types that are stable at ambient temperature, creating cost-effective supply chains to facilitate logistics of samples, and the development of a smartphone-enabled portal for data entry and analyses are among the quality assurance measures that can improve the quality and accuracy of testing, reduce errors, and increase utilization of POC tests.

## REMAINING ISSUES WITH HCV DIAGNOSTIC DOWN-CLASSIFICATION

The down-classification of HCV diagnostics (antibody and HCV RNA tests) offers real hope for more diagnostic options in the US. It is hoped that this will promote the necessary steps to get current anti-HCV and HCV RNA POC testing into broader use and spark innovation by diagnostic companies to further improve tests. For example, the POC diagnostics for HCV RNA (eg, GenXpert HCV RNA assay) has a turnaround time of approximately 1 hour [14], which is suboptimal for a test-and-treatment model of care. The ideal POC test would offer a confirmation of diagnosis with a turnaround time of 20 minutes or less [15].

An area of uncertainty pertains to whether the hepatitis C virus core antigen test (HCVcAg) will be eligible for down-classification or not. HCVcAg correlates well with HCV RNA levels ≥10 000 IU/mL and, thus, can detect viremia in the vast majority of persons with active HCV infection. A recent metanalysis of 46 studies using HCVcAg reported the assay to have high specificity and sensitivity for active HCV infection but suboptimal positive predictive value in low prevalence settings [16]. Nonetheless, this test may be a valuable addition to the rapid diagnostic armamentarium, especially in high-prevalence settings where nucleic acid testing is less feasible or unavailable [15]. Yet, the designation of class III versus class II tests will likely influence the timeline for HCVcAg tests being integrated into future screening algorithms.

One final note regarding the FDA recent down-classification for HCV tests from class III to class II is that it does not include over-the-counter diagnostic tests, something the public has come to embrace under COVID-19. The FDA stated that a nucleic acid–based HCV RNA test intended for over-the-counter use would be a new type of device not previously classified and, as a result, these postamendment devices would be automatically classified into class III [17]. Consequently, over-the-counter tests for HCV are unlikely to be available soon.

## LOOKING FORWARD: HCV DIAGNOSTICS IN THE FUTURE

As highighted, there is hope that down-classification of antibody and HCV RNA diagnostics will facilitate innovation and contribute to simplified diagnostic algorithms. Technologic advances, driven in part by the emerging global infectious disease threats (severe acute respiratory syndrome coronavirus 2, Ebola virus, and others) are yielding tests that are accurate, have rapid turnaround, and are scalable to large numbers of tests [18]. Similarly, to advance global HCV elimination goals, it is essential to have rapid diagnostic tests applicable to a range of clinical settings. Innovative devices for detecting HCV in serum or plasma include loop-mediated isothermal amplification, smartphone-operated instruments, nanoparticle- and microchip-based systems such as microfluidic and microarray platforms, and those using CRISPR-Cas technology [19, 20]. A particular advantage of CRISPR-Cas technology is the ability of all the enzymes to work at low temperatures, obviating the need for special equipment for nucleic acid amplification. A recent study comparing detection of HCV RNA based on a reverse-transcription loop-mediated isothermal amplification–coupled CRISPR-Cas12 to the reference method (Roche COBAS AmpliPrep/Cobas TaqMan HCV test) in clinical samples from individuals infected with HCV, HIV, or hepatitis B virus, or healthy controls, reported that the CRISPR-Cas12 method had 96% sensitivity, 100% specificity, and 97% agreement [21]. This serves as just one example of how innovative platforms can be expected to change the landscape of HCV diagnostics in the near future. With increasing use of new platforms, it is hoped that FDA regulations will continue to evolve to support and enable diagnostic advancements for HCV infection.

## Notes

***Supplement sponsorship.*** This article appears as part of the supplement “Towards a Single-Step Diagnosis of Hepatitis C Virus Infection,” sponsored by the Centers for Disease Control and Prevention.
